# Cardiopulmonary bypass versus non-bypass surgery for tumor thrombus extending into the inferior vena cava or right atrium in non-cardiac malignancies: a systematic review and meta-analysis

**DOI:** 10.1186/s12957-025-03956-2

**Published:** 2025-10-14

**Authors:** Ahmed Dawood Al Mahrizi, Fatima Mossolem, Fatima Zahra Achaq, Erin Major, Moiuz Chaudhri, Arthur Okere

**Affiliations:** 1 Faculty of Medicine & Surgery, University of Malta, Msida, Malta; 2 Futures Forward Research Institute, Toms River, NJ USA; 3 Hackensack Meridian Ocean University Medical Center, Brick Township, NJ USA; 4 Faculty of Medicine and Pharmacy of Rabat, University of Mohammed 5, Rabat, Morocco

**Keywords:** Surgical management, Tumor thrombus, Inferior vena cava, Right atrium, Noncardiac malignancies, Cardiopulmonary bypass

## Abstract

**Background:**

The role of cardiopulmonary bypass (CPB) in surgery for tumor thrombi extending into the inferior vena cava (IVC) or right atrium in noncardiac malignancies remains controversial. We systematically compared perioperative mortality and complications between the CPB and non-CPB approaches and summarized findings on surgical techniques and patient selection.

**Methods:**

We searched major databases for studies (2015–2025) reporting surgical resection of IVC or right atrial tumor thrombi in noncardiac malignancies stratified by CPB use. Twenty studies were included in the qualitative synthesis, and nine were included in the quantitative meta-analysis. Pooled perioperative mortality and complications were compared via random effects models. Heterogeneity and correlations between CPB use and mortality were assessed.

**Results:**

Surgical approaches varied. CPB was typically reserved for extensive thrombus or right atrial involvement. The complication and recurrence rates were heterogeneous. In nine studies (800 patients), the pooled perioperative mortality rate was 9.2% (95% CI: 4.7–17.1%), with no significant difference between the CPB (10.3%) and non-CPB (7.8%) groups. High heterogeneity reflected differences in patient selection, tumor extent, and surgical technique. Qualitative synthesis indicated that CPB increased procedural complexity and risk but was utilized for more advanced disease. Recurrence and long-term outcomes have been inconsistently reported. Most studies were retrospective and at moderate to serious risk of bias.

**Conclusion:**

Surgical management with or without CPB results in similar perioperative mortality rates. CPB is generally reserved for extensive thrombi, adding complexity and risk. Individualized, multidisciplinary planning is essential. Prospective studies are needed to refine patient selection and optimize surgical strategies in this challenging setting.

**Supplementary Information:**

The online version contains supplementary material available at 10.1186/s12957-025-03956-2.

## Introduction

Cardiopulmonary bypass (CPB) plays a critical role in the surgical management of complex renal tumor thrombi, particularly in cases where the thrombus extends into the inferior vena cava (IVC) or right atrium [1]. Surgical intervention is often necessary because of the high risk of complications associated with tumor thrombus, including sudden death from thrombus migration or extension, which can lead to severe cardiovascular events [2, 3].

Patients typically present with symptoms such as respiratory distress, abdominal pain, or signs of venous obstruction, necessitating urgent surgical intervention to improve survival outcomes [4, 5]. The surgical approach can be challenging, with major complications and mortality risks, particularly when CPB is utilized, which is associated with complications such as coagulopathy and prolonged operative times [6].

The complications arising from open surgical repair of tumor thrombi can be substantial and include severe bleeding, pulmonary embolism, and duodenal perforation [7]. Minimally invasive techniques are being explored as alternatives to traditional approaches, potentially reducing the incidence of these complications while maintaining effective tumor removal [8]. These strategies aim to enhance patient recovery and minimize the risks associated with CPB, offering a promising avenue for improving surgical outcomes in patients with complex tumor thrombi [6]. However, the choice of surgical strategy must be tailored to the individual patient's condition and tumor characteristics to optimize the results [9].

The use of CPB, often combined with deep hypothermic circulatory arrest (DHCA), facilitates the safe resection of tumor thrombi by providing a bloodless surgical field and reducing the risk of embolization and severe hemorrhage [10–12]. Studies have shown that CPB can be employed effectively in both minimally invasive and traditional surgical approaches, with the choice of technique often depending on the thrombus level and patient-specific factors [13, 14]. For example, minimally invasive techniques without sternotomy have been developed to reduce surgical trauma and improve recovery times, although these require careful patient selection and expertise [14].

In 2005, Ciancio and Soloway first reported a purely transabdominal approach for 59 patients with level III–IV IVC thrombi, achieving complete resection without sternotomy or CPB; only 5% required bypass and perioperative mortality was 4.4%. In a 2010 cohort of 12 patients with supradiaphragmatic and intra-atrial thrombi, Ciancio et al. demonstrated that a transdiaphragmatic incision with piggyback liver mobilization and suprahepatic/infrarenal IVC clamping permitted safe tumor extraction without CPB or DHCA and without intraoperative embolic events. A subsequent step-by-step description in 68 patients confirmed the reproducibility of this liver-transplant-style technique, reporting 7.3% CPB use and 4.4% immediate postoperative mortality [15–17].

Surgical management with CPB has been discussed specifically in the context of renal cell carcinoma (RCC) tumor thrombus management, but its value remains understudied, with few studies being able to present succinct management algorithms [18, 19]. Renal cell carcinoma with a tumor thrombus is relatively uncommon but can prove difficult to manage surgically [20]. The tumor thrombus in these patients can extend from the kidney parenchyma through the lumen of the inferior vena cava to the right atrium [21]. The extension of tumor margins through these tissues makes surgical management difficult so as not to disrupt cardiopulmonary circulation. Surgically accessing the IVC and right atrium to remove the tumor could compromise venous return and circulation overall, thus explaining why CPB may be useful in this scenario [22]. The use of CPB allows the surgical team to resect tumor margins in the IVC and right atrium while the patient's oxygenation and perfusion status is maintained on bypass [22]. While in theory, the use of bypass should provide surgical outcomes, some studies have shown that CPB can negatively impact survival, with CPB patients having lower median survival [23]. In contrast, other studies have shown that CPB is valuable in reducing operative blood loss and provides comparable long-term survival rates [11, 24]. The varying effects of CPB in patients with RCC tumor thrombus highlight the need for a review to better understand the role of this treatment modality. This review presents a succinct summary of the associated outcomes of patients who underwent surgical resection of RCC with and without cardiopulmonary bypass assistance. Despite numerous single-center reports, there remains no consensus on the optimal surgical approach for noncardiac malignancies in which the tumor thrombus extends into the IVC or right atrium. This systematic review and meta-analysis synthesizes the most recent evidence, directly comparing outcomes with and without CPB, to inform evidence-based surgical decision-making and highlight areas for future research.

## Methods

This systematic review and meta-analysis were performed in line with the Preferred Reporting Index for Systematic Reviews and Meta-analyses (PRISMA 2020) guidelines. This study was also registered on PROSPERO (CRD420251035345) [25]. The protocol and inclusion criteria for this study were defined a priori through the use of the PICO framework.

### Search methodology and study inclusion criteria

A systematic search was conducted on several databases, including PubMed, Embase, Scopus, Web of Science, and Google Scholar, from January 2015 to April 2025. The search strategies involved terms related to tumor thrombus, the inferior vena cava, the right atrium, surgical resection, and cardiopulmonary bypass and were adapted for each database (Supplementary Annex S1). References of included studies and relevant reviews were also screened.

Two independent reviewers screened titles and abstracts for eligibility, with conflicts resolved by discussion. Full-text analysis was further performed by two independent reviewers with inter-rater agreement. The inclusion criteria were as follows: (1) adults (≥ 18 years) with noncardiac malignancy and radiologic or surgical confirmation of a tumor thrombus extending into the IVC (level III/IV — Level III: thrombus extends to the hepatic veins; Level IV: thrombus extends into the right atrium) [26] or right atrium; (2) surgical management with or without CPB; (3) reporting of at least two relevant outcomes (mortality, complications, recurrence, or follow-up); and (4) cohort size ≥ 5. The exclusion criteria were as follows: cardiac tumors, pediatric cases, studies published before 2015, case reports, studies published in languages other than English, and studies with unclear CPB status. Discrepancies were resolved by consensus. We were limited to English-language publications due to resource constraints; however, grey-literature searches in Google Scholar and reference lists mitigated language bias.

### Extraction of data and quality assessment

The data was independently extracted by two reviewers through a standardized template capturing different study parameters, such as tumor pathology, thrombus levels, use of cardiopulmonary bypass (including binary values or percentages), surgical methods, mortality, complication rates, rates of disease recurrence, and follow-up duration. Qualitative information on surgical methods, complication profiles, and decision-making factors was also recorded. The two reviewers extracted the data separately, where discrepancies were resolved via consensus. The qualitative synthesis was based on 20 studies, while 9 studies contained adequate data for quantitative meta-analysis.

### Outcome

The primary outcome was perioperative or in-hospital mortality. The secondary outcomes included major complications, recurrence, and follow-up.

### Integration with data and evaluative statistics

A random-effects meta-analysis of proportions was performed via the inverse variance method with logit transformation and the DerSimonian‒Laird estimator for between-study variance. We selected the DerSimonian–Laird estimator for τ^2^ because it is robust for proportional data with moderate study counts and is standard in surgical meta-analyses [27]. Subgroup analysis compared the CPB and non-CPB cohorts. Heterogeneity was quantified with I^2^ and τ^2^ statistics. Funnel plots were generated to assess publication bias. The correlation between percent CPB use and mortality was evaluated via Pearson’s correlation coefficient. Qualitative synthesis was conducted for all included studies, summarizing surgical approaches, complication patterns, and patient selection criteria. We conducted a leave-one-out sensitivity analysis by iteratively excluding each study and re-running the random-effects meta-analysis to assess the influence of individual cohorts on the pooled mortality estimate and heterogeneity.

**Fig. 1:**
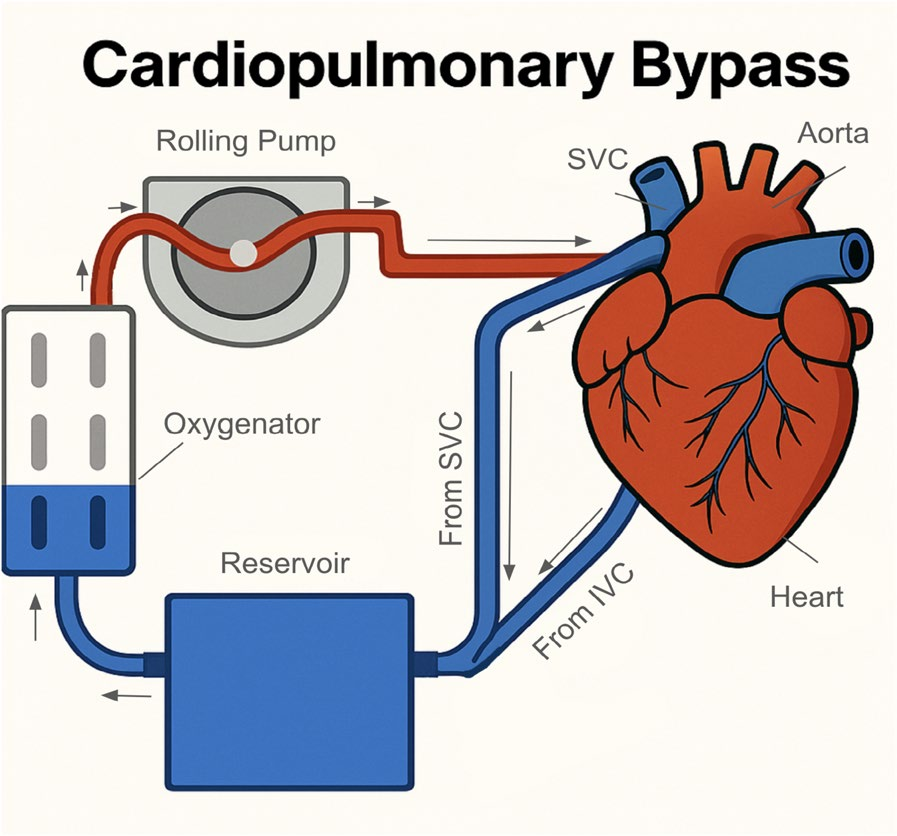
Schematic diagram illustrating the components and flow of a cardiopulmonary bypass circuit, including venous drainage from the superior and inferior vena cava (SVC, IVC), reservoir, oxygenator, rolling pump, and arterial return to the aorta. Figure created by Ahmed D. Al Mahrizi, one of the study authors

**Fig. 2:**
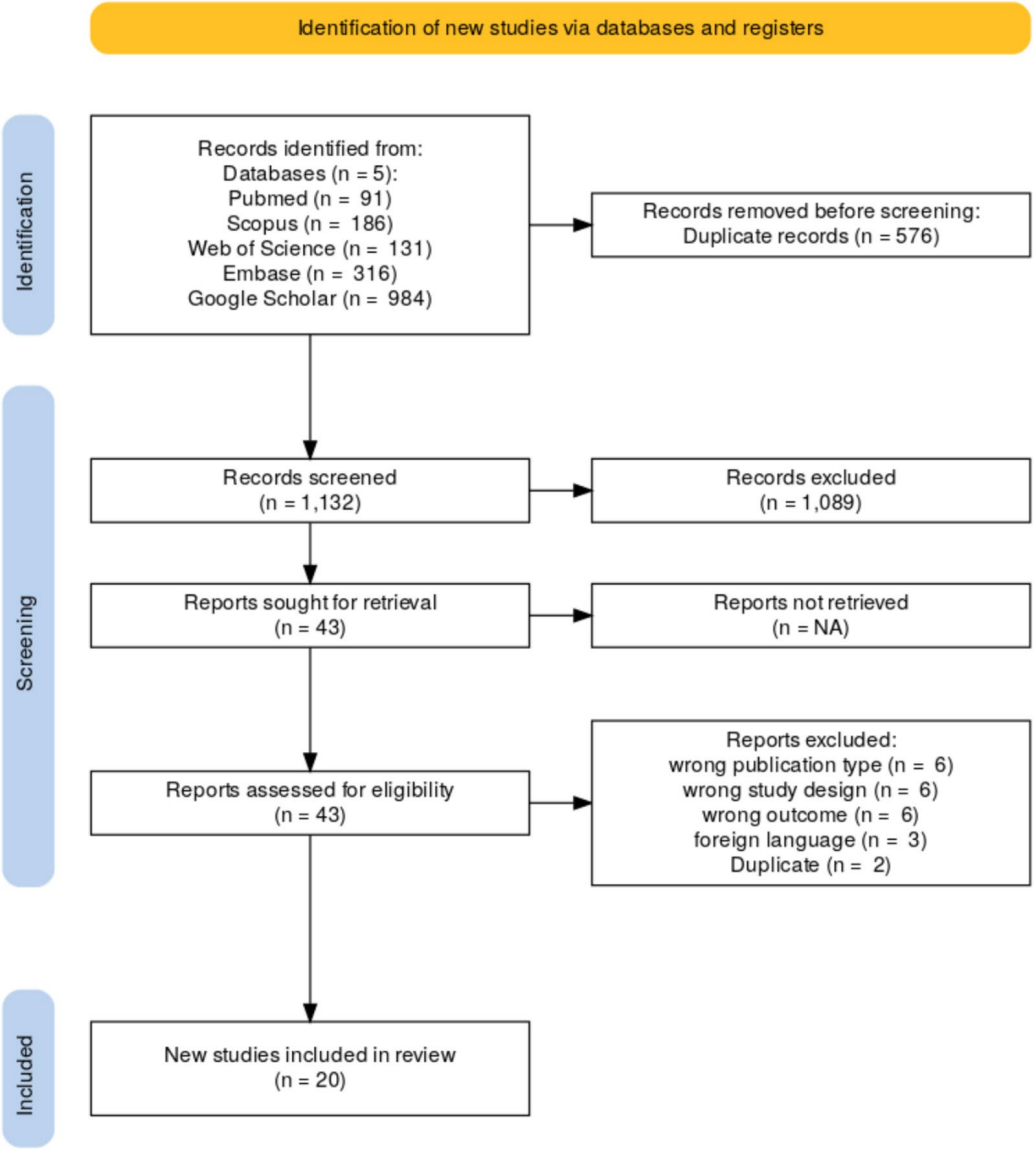
PRISMA flow diagram of study selections

All analyses were performed via R programming with the meta and dplyr packages [28, 29]. The PRISMA flow diagram (Figs. 1 and 2) explains how the studies were selected.

### Risk of bias

The risk of bias in this review was evaluated by two independent reviewers via a modified Risk of Bias in Non-Randomized Studies of Interventions (ROBINS-IV2) framework adapted for retrospective observational studies. The two reviewers independently applied a modified ROBINS-I V2 tool to each study, with adjudication of domain conflicts through consensus and, when necessary, consultation with a third reviewer. Additionally, the certainty of evidence for each outcome was assessed via the GRADE approach, which incorporates risk of bias, inconsistency, indirectness, and publishing bias to rate the overall confidence in effect estimates across studies.

## Results

### Study selection and characteristics

A total of 1,708 unique records were gathered through systematic searches of databases such as PubMed, Embase, Scopus, Web of Science, and Google Scholar. After deduplication and screening, 20 studies published from 2015 to 2025 were chosen for qualitative synthesis, and 9 studies involving 800 patients and 99 deaths provided sufficient quantitative data for meta-analysis. The studies included were comprised of retrospective analyses, multicenter questionnaires, and a single prospective observational study, with varying tumor types (e.g., renal cell carcinoma, hepatocellular carcinoma, adrenal cortical carcinoma, sarcoma, etc.) and thrombus grades (III/IV and extension to the right atrium) covered. The use of CPB was variable, ranging from 0 to 100%, with most studies describing stratified or mixed research types.

### Qualitative analysis

Surgical techniques included midline laparotomy, thoracoabdominal incisions, sternotomy, and minimally invasive procedures. CPB was largely reserved for extensive thrombi in the IVC or right atrium, with some studies applying deep hypothermic circulatory arrest (DHCA) to improve exposure and reduce embolic risk. The use of CPB was determined by the size of the thrombus, the comorbid state of the patient, and institutional expertise. In clinical practice, CPB is typically reserved for patients with Level IV thrombi or those with right-atrial extension in whom safe vascular control cannot be achieved by IVC cross-clamping alone. This approach balances the potential reduction in embolic and bleeding risk against the well-documented morbidities of CPB, including coagulopathy, neurologic injury, and systemic inflammation. The complication profiles were heterogeneous; major complications included hemorrhage, renal failure, cardiovascular events, neurological injury, and infection. Some studies reported high rates of specific complications (e.g., renal failure, prolonged ventilation, wound infection, and sepsis) in the CPB group. Recurrence rates and long-term survival rates varied, with follow-up times ranging from 1.2 to 46.5 months. Long-term oncologic outcomes (disease-free and overall survival) were reported inconsistently (median follow-up ranged from 1.2–46.5 months), precluding meta-analysis of durability and limiting recommendations for long-term decision-making. Many studies have emphasized the indispensable role of interdisciplinary cooperation and the need for individualized surgical planning.

### Quantitative analysis

#### Pooled mortality

**Fig. 3:**
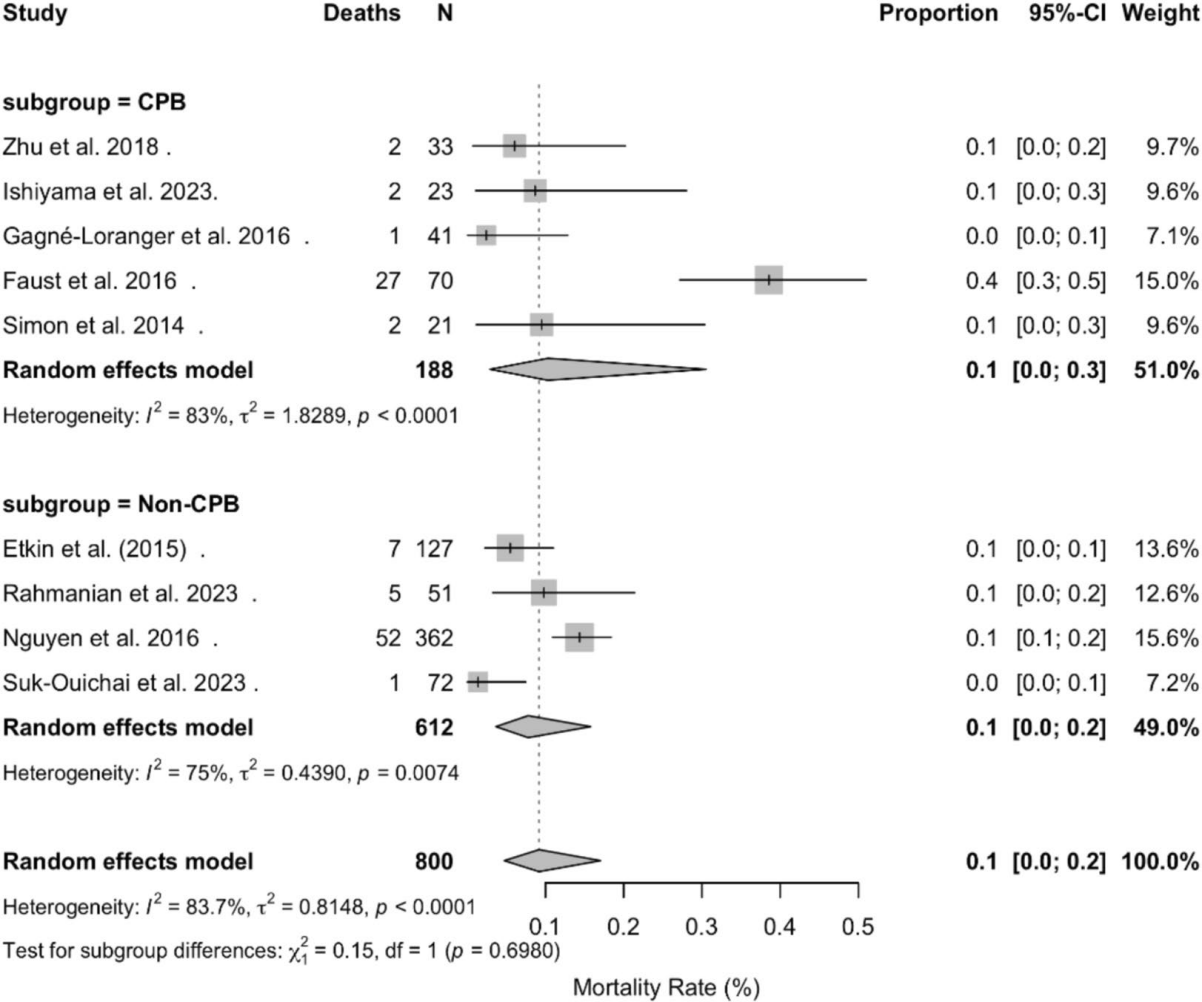
Forest plot of pooled and subgroup mortality rates for the CPB and non-CPB cohorts

A meta-analysis involving a total of nine studies with 800 patients demonstrated an overall perioperative mortality rate of 9.2% (95% CI: 4.7–17.1%) (Fig. 3). The subgroup analysis for CPB and non-CPB use reported a mortality rate of 10.3% (95% CI: 2.9–30.5%; I^2^ = 83%) for CPB patients and 7.8% (95% CI: 3.7–15.8%; I^2^ = 75%) for non CPB patients. The comparison across groups was not statistically significant (Q₁ = 0.15, *p* = 0.698) and thus reflects no significant mortality benefits or limitations with the use of CPB.

The studies had considerable heterogeneity (total I^2^ = 83.7%, *p* < 0.0001), reflecting real-world variability in patient and tumor characteristics, surgical expertise, and institutional protocols. This underscores the challenge of standardizing surgical management and the importance of individualized treatment planning.

#### Publication bias

**Fig. 4:**
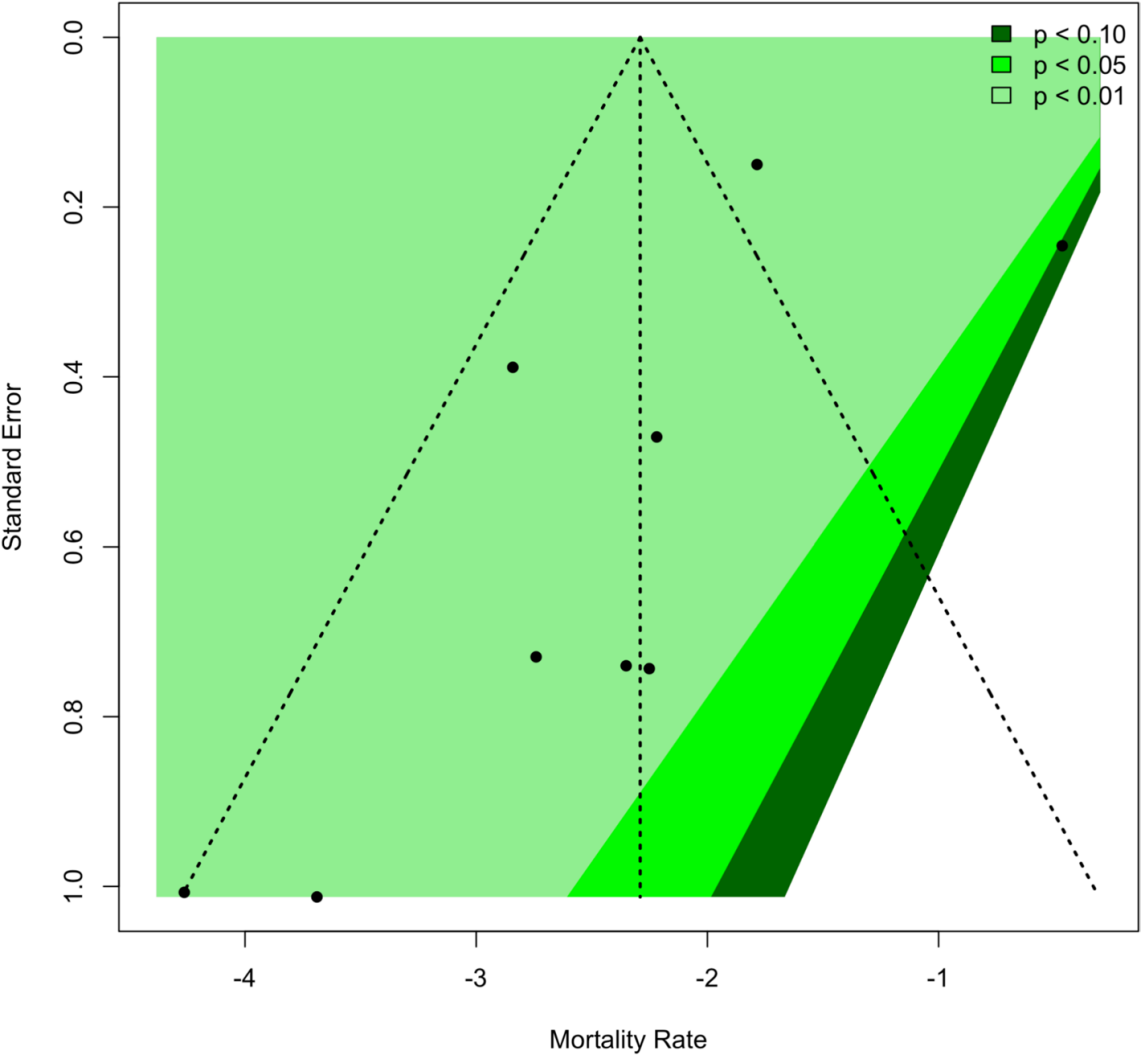
Funnel plot for publication bias assessment

Funnel plot analysis (Fig. 4) revealed satisfactory symmetry among studies with no major indication of serious publication bias, although this low number of studies limits formal assessment. Given the small number of studies, small-study effects cannot be excluded and may have influenced the symmetry observed.

#### Relationship between CPB utilization and mortality rates

**Fig. 5:**
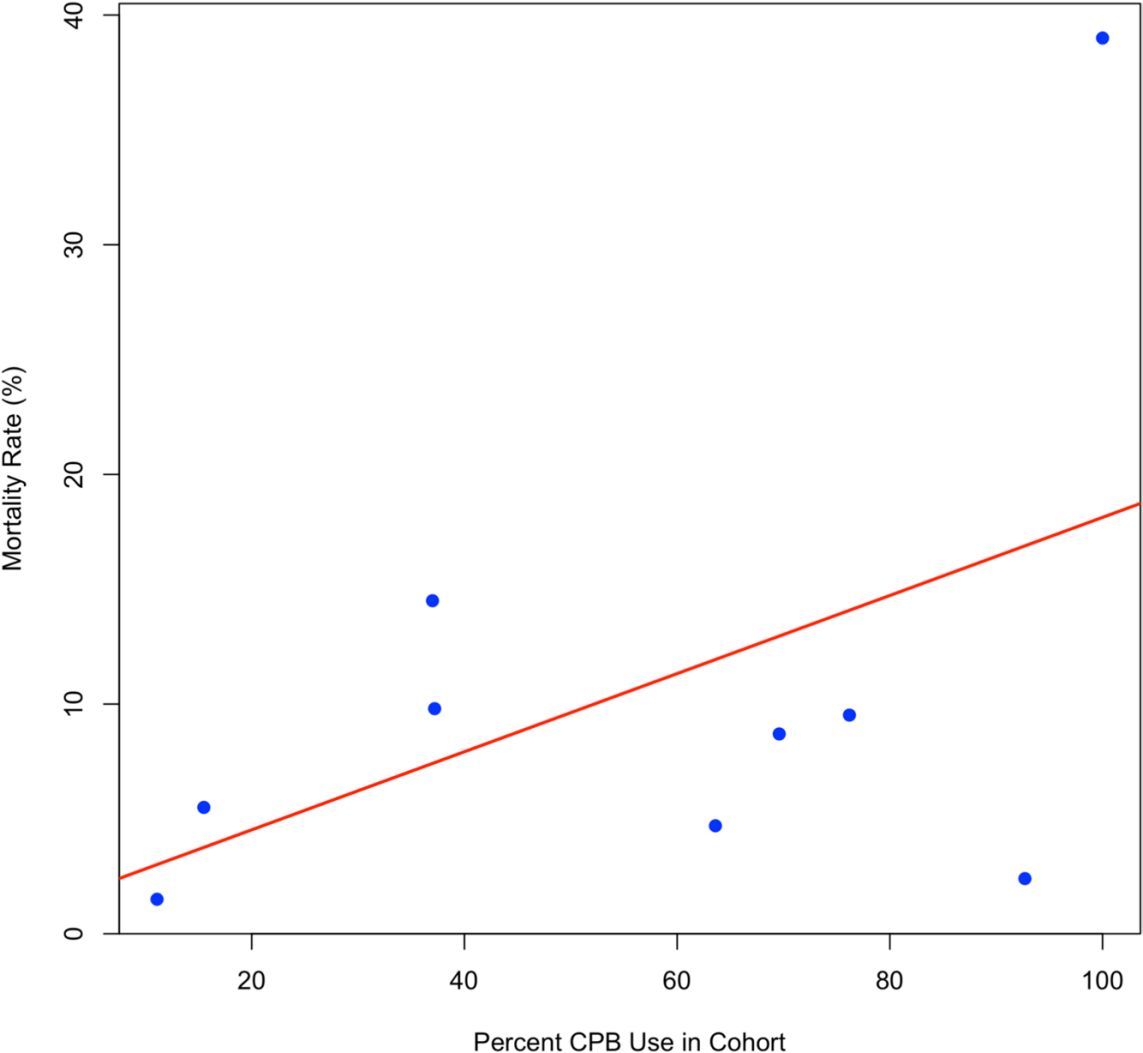
Scatter plot showing the relationship between the percentage of CPB use and the mortality rate

A mild, nonsignificant positive correlation existed when mortality rates were plotted against the incidence of CPB use among the studied cohorts (Pearson’s r = 0.48, 95% CI: –0.27 to 0.87, *p* = 0.19; Fig. 5). This finding reflects an underlying trend toward higher mortality in cohorts with high CPB use; however, it is important to note that this finding was not statistically significant (Table 1).

**Table 1:** Summary of Findings Table

Study (Year)	N	Mortality (%)	Major Complications (%) (95% CI)	Recurrence (%)	Follow-up (mo)	Comments
Zhu et al. 2018 [30]	33	4.7	12.1% (95% CI 3.4–28.2)	NR	22	Radical nephrectomy + CPB/DHCA via sternotomy
Xiao et al. 2017 [31]	103	2.9	18.8% (95% CI 12.1–27.0)	NR	46	20-year single-center experience, transabdominal approach
Ishiyama et al. 2023 [32]	23	8.7	6.3% (95% CI 0.2–27.0)	70.6%	1.2	Sternotomy + CPB for right-atrial thrombus; grade ≥ 3 complications
Etkin et al. 2015 [33]	127	5.5	5.5% (95% CI 2.7–10.9)	NR	NR	Mixed tumor types, midline/thoracoabdominal approaches
Gagné-Loranger et al. 2016 [26]	41	2.4	2.4% (95% CI 0.4–12.6)	NR	22.8	CPB + DHCA for level III/IV; axillary cannulation
Rahmanian et al. 2023 [34]	51	9.8	5.8% (95% CI 2.0–15.9)	NR	46.5	Laparotomy + sternotomy; 37.2% CPB use
Vinzant et al. 2022 [35]	65	6.8	41.2% (95% CI 30.4–53.7)	NR	NR	Level III/IV thrombi; stratified CPB vs VVB comparison
Morita et al. 2016 [36]	NR	25.0	NR	NR	NR	Case series; CPB ± DHCA anesthesia management
Carmignani et al. 2024 [37]	12	33.3	NR	NR	27.5	Beating-heart normothermic CPB for level III tumors
Ichida et al. 2025 [38]	119	NR	NR	NR	NR	Multicenter survey of HCC thrombi resection
Faust et al. 2016 [39]	70	39.0	30.4% (95% CI 20.5–41.5)	41.5% (MS) 16.7% (MA)	NR	MS vs minimal-access CPB; higher renal failure & sepsis in MS group
Issard et al. 2021 [40]	72	8.0	NR	75%	15.1	Group III/IV: 77% CPB, 17% DHCA
Dashkevich et al. 2016 [41]	35	NR	17.0% (95% CI 8.1–32.7)	31%	NR	Midline laparotomy + CPB/DHCA for level IV
Simon et al. 2014 [42]	21	9.5	31.3% (95% CI 13.9–54.9)	NR	11.9	CPB vs VVB comparison; radical nephrectomy
Chao et al. 2022 [43]	24	NR	NR	71.4%	12.0	Staged vs concomitant hepatectomy + thrombectomy
Cai et al. 2022 [44]	65	NR	11.5% (95% CI 5.3–20.6)	NR	6.0	Prospective observational; CPB + DHCA in level III/IV
Öbius et al. 2022 [45]	24	9.1	NR	NR	NR	Chevron-incision CPB; mild vs moderate hypothermia
Nguyen et al. 2016 [46]	362	14.4	27.0% (95% CI 22.8–31.9)	NR	13.8	Multi-institutional, CPB vs non-CPB
Suk-Ouichai et al. 2023 [23]	72	1.5	50.0% (95% CI 38.7–61.3)	NR	19.7	Higher complication rate in CPB vs non-CPB cohorts
Chen et al. 2015 [47]	32	6.3	12.5% (95% CI 3.5–29.2)	33.3%	25.0	Minimally invasive CPB + DHCA for level III/IV

^†^ N = sample size; NR = not reported; MS = median sternotomy; MA = minimal access

^‡^ Major complications defined as Clavien–Dindo grade III–V events

### Risk of bias in studies

**Fig. 6:**
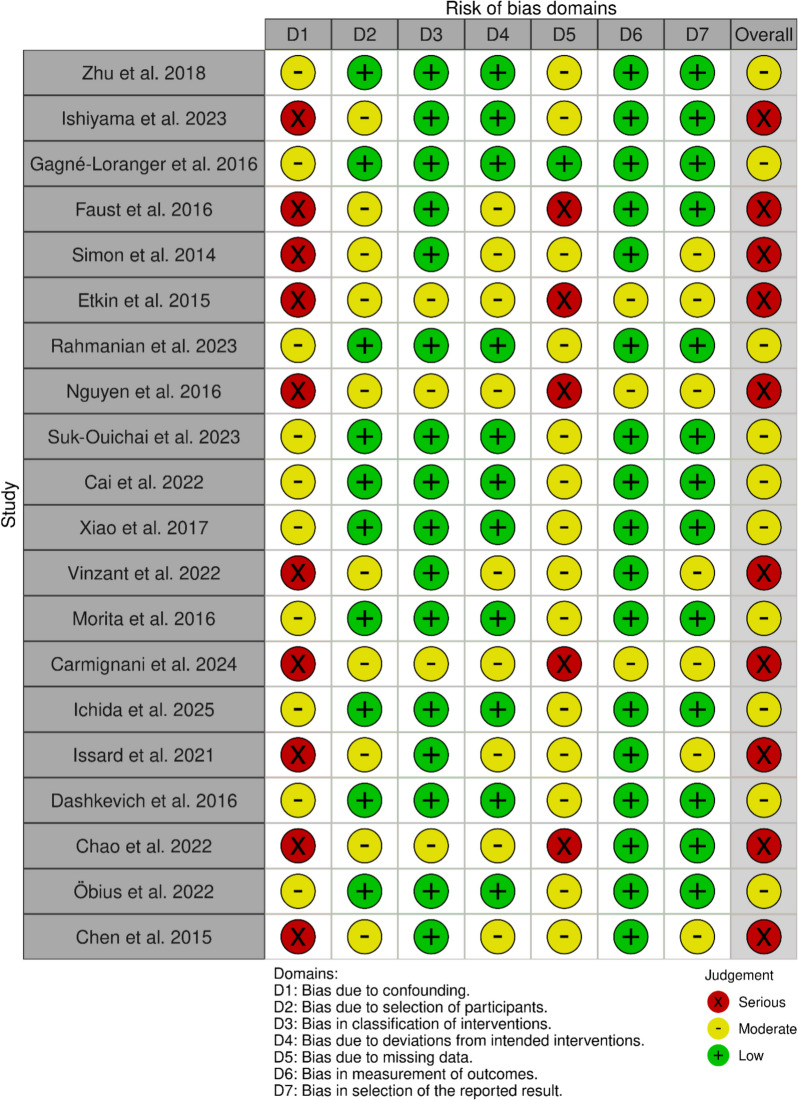
Risk of bias visualized in a traffic light plot

**Fig. 7:**
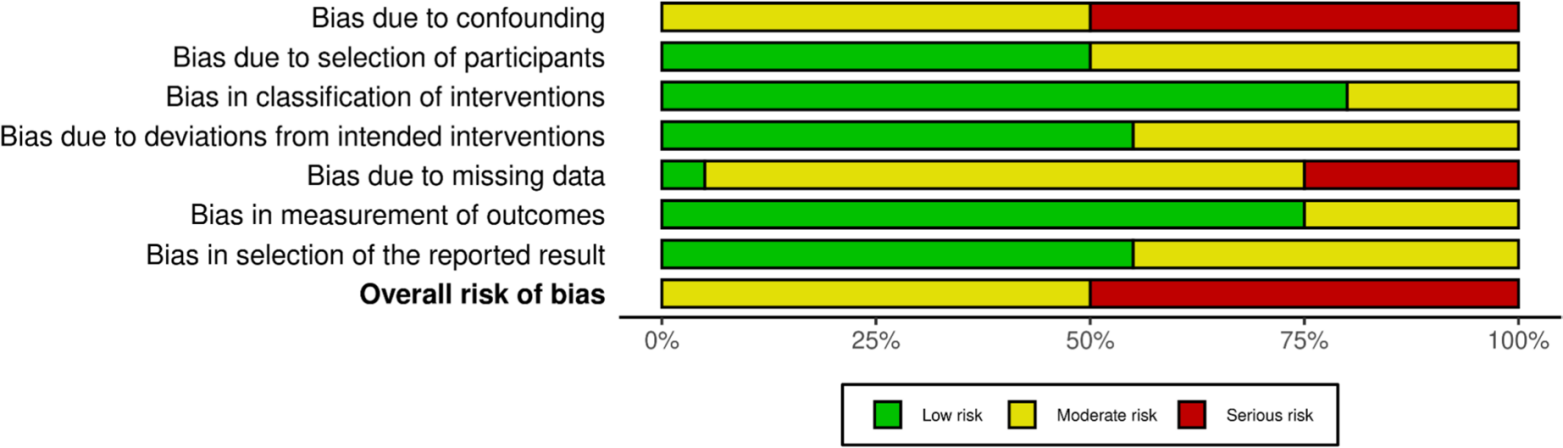
Summary plot for risk of bias

The risk of bias was evaluated in all 20 studies included in this analysis via the ROBINS-I V2 instrument, adapted for use with retrospective observational cohorts. Most studies had a moderate or serious overall risk of bias. The most frequent sources of bias were confounding variables due to inadequate adjustment for important prognostic factors such as tumor load, comorbidities, and surgeon experience, as well as missing data due to incomplete outcome reporting or loss to follow-up. The risk of selection bias was mostly classified as low to moderate, as most cohorts included consecutive or all eligible patients; however, some studies reported unclear or restrictive inclusion criteria. The grouping of interventions was mostly adequately defined, with most having a low risk, although a few studies had mixed or unclear groupings. The risk of bias due to deviation from intended interventions was mostly low, although some studies reported little information about protocol adherence. Outcome assessment mostly has a low risk due to mortality and major complication outcome associations with objectivity, whereas risk due to selective reporting ranges from low to moderate depending on protocol registration and outcome completeness. Overall, these limitations are inherent to retrospective operational series and should be interpreted with care when looking at aggregated estimates (Figs. 6 and 7).

### Certainty of evidence

Confidence regarding the primary outcome was rated as low after the application of the GRADE framework. This rating reflects the predominance of multiple nonrandomized, retrospective studies that have a high or moderate risk of bias, as ascertained by the ROBINS-I V2 assessment, with major discrepancies characterized by high levels of heterogeneity (I^2^ > 75%) among the studies examined. Moreover, a high level of imprecision was noted in terms of wide confidence intervals and relatively low rates of events, whereas indirectness was minimal since the included studies directly addressed the population, interventions, and outcomes. Although the funnel plot analysis did not identify publication bias, the small number of studies limits how much such conclusions may definitively be drawn regarding such bias. Collectively, these factors require careful interpretation of pooled estimates and support future high-quality prospective studies intended to improve the reliability and size of these effect estimates.

### Sensitivity analysis

**Fig. 8:**
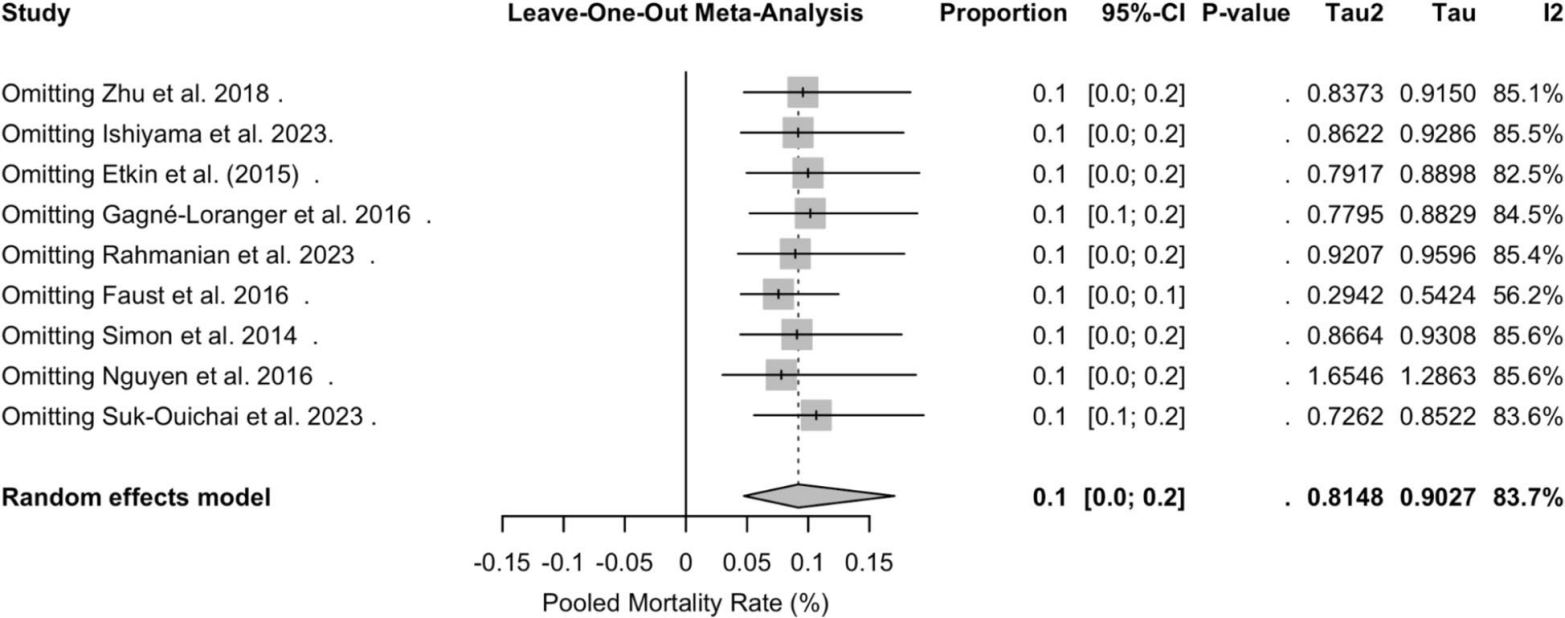
Leave-one-out sensitivity analysis forest plot of pooled perioperative mortality

The leave-one-out analysis demonstrated that omission of any single study altered the overall pooled mortality by no more than ± 1.2 percentage points and did not materially change between-study heterogeneity (I^2^ remained above 82%). Excluding Faust et al. (2016) had the greatest impact, reducing the pooled mortality from 9.2% to 7.6% and lowering I^2^ from 83.7% to 56.2% [39]. These findings confirm that no individual study unduly drives the meta-analytic results, supporting the robustness of our pooled estimates (Fig. 8).

## Discussion

### Mortality

Overall, our analysis revealed no significant differences in mortality between CPB users and non-CPB users, despite CPB generally being used for more extensive disease processes and not as a first-line treatment for general pathology [1]. This review is the first in the past decade to systematically compare the perioperative outcomes of CPB and non-CPB approaches in a broad spectrum of noncardiac malignancies, providing a comprehensive synthesis for surgical oncologists. Our analysis revealed an overall perioperative mortality rate of 9.2% (95% CI: 4.7–17.1%) among both groups, emphasizing the severity of disease necessary for patients to even be considered for CPB [48]. Importantly, the subgroup analysis yielded a mortality rate of 10.3% (95% CI: 2.9–30.5%; I^2^ = 83%) for CPB users and 7.8% (95% CI: 3.7–15.8%; I^2^ = 75%) for nonusers; however, the difference in mortality was not statistically significant. Patients who underwent CPB were generally those for whom less invasive cardiopulmonary procedures were not suitable, and some form of open heart surgery was required—consistent with clinical guidelines [49]. Further analysis also revealed a nonsignificant positive correlation between mortality and CPB (Pearson’s r = 0.48, 95% CI: −0.27 to 0.87, *p* = 0.19), which demonstrated increased mortality in cohorts with high CPB use. However, because this finding is not significant, it is important to remember that CPB is not used first-line in many cardiac malignancies and is primarily reserved for extensive disease processes [50, 51]. While many non-CPB surgical procedures are used in accordance with each patient's unique characteristics, we found that the use of CPB was determined mainly by the size of the thrombus with the patient’s comorbidities and institutional expertise, which also play a significant role.

Substantial heterogeneity was observed across studies (I^2^ = 83.7%, *p* < 0.0001). To determine whether any single cohort unduly influenced the pooled mortality estimate, we conducted a leave-one-out sensitivity analysis. Omitting Faust et al. (2016) reduced I^2^ to 56.2% and lowered the pooled mortality from 9.2% to 7.6%, whereas exclusion of each other study shifted the estimate by no more than ± 1.2 percentage points and maintained I^2^ above 82% [39]. These findings indicate that no single study drives the overall result and that the high heterogeneity likely reflects true variation in patient selection, thrombus extent, and surgical technique rather than an outlier effect [1]. The surgical techniques used in comparison with CPB varied significantly. This finding is important to highlight, as it further demonstrates that even with a wide variety of techniques, mortality rates between the two groups did not vary significantly. Studies employing minimally invasive or organ-sparing approaches without CPB, such as Guglielmo et al.’s step-by-step technique [19], reported lower median blood loss and shorter ventilation times, suggesting technique selection may influence perioperative morbidity independent of CPB use. The complication profiles also proved to be heterogeneous. Many studies have reported major complications, such as hemorrhage, renal failure, cardiovascular events, neurological injury, and infections. These rates seemed to vary among the CPB groups and non-CPB groups; however, we did observe more reports of complications such as renal failure, prolonged ventilation, wound infection, and sepsis in the CPB groups. These adverse events can arise from a combination of factors consistent with the mechanism of CPB [52]. CPB triggers a systemic inflammatory response within a patient due to contact with artificial surfaces [53]. Ischemia‒reperfusion injury and surgical trauma are also common due to the nature of the disease process [54]. This emphasizes the need to utilize an interdisciplinary team and individualized surgical planning to ensure the best outcomes for each patient, with many studies emphasizing the need for collaboration in patient care [9]. Given the lack of a clear mortality benefit and the increased complexity associated with CPB, surgical teams should weigh the risks and benefits on a case-by-case basis, considering tumor extent, patient comorbidities, and institutional experience. These findings support a patient-centered, multidisciplinary approach rather than a uniform protocol.

### Limitations

Limitations of this review include the predominance of retrospective studies, moderate to serious risk of bias, and substantial heterogeneity among the included cohorts. These factors limit the certainty of pooled estimates and highlight the need for prospective, controlled studies to refine patient selection and surgical strategies. Moreover, the retrospective design of all included cohorts and moderate-to-serious ROBINS-I V2 ratings weaken causal inferences, and findings should be interpreted with caution.

### Future directions

Due to the lack of statistically significant differences in mortality between CPB patients and non-CPB patients, future studies should focus on mortality in patients with similar tumor progression rates who undergo surgical resection. This would help guide clinicians in determining when CPB is the best option in regard to surgical care.

### Long term outcomes

A subset of six studies (of the 20 qualitatively synthesized) reported a median follow-up of at least 24 months and provided explicit long-term survival data. In these cohorts, 2-year overall survival ranged from 40 to 70%, with no consistent difference between CPB and non-CPB groups. Heterogeneity in follow-up duration, surveillance protocols, and outcome definitions prevented quantitative pooling of these long-term outcomes. These findings emphasize the need for future prospective studies with standardized, long-term endpoint reporting to determine the durability of CPB versus non-CPB approaches.

## Conclusion

This systematic review and meta-analysis revealed an overall perioperative mortality of 9.2% (95% CI, 4.7–17.1%), with no significant difference between the CPB cohort (10.3%) and the non-CPB cohort (7.8%; *p* = 0.698), despite CPB being reserved for more extensive thrombus burden. Marked heterogeneity emphasizes the importance of individualized, multidisciplinary planning in surgical oncology. CPB should be reserved for select patients with extensive thrombi, with careful consideration of patient and tumor factors. This review provides up-to-date evidence to guide surgical strategy selection. However, substantial heterogeneity in complication and recurrence rates, the moderate-to-serious risk of bias inherent to predominantly retrospective studies, and inconsistent reporting of long-term survival temper the strength of these conclusions. These factors highlight the need for prospective, controlled trials with standardized outcome reporting to better define optimal surgical strategies.

## Supplementary Information

Supplementary Material 1.

Supplementary Material 2.

## Data Availability

All data generated or analyzed during this study are included in this published article and its supplementary information files. The data extraction sheet is available upon reasonable request from the corresponding author.

## Abbreviations

CPB: Cardiopulmonary bypass
IVC: Inferior vena cava
RA: Right atrium
DHCA: Deep hypothermic circulatory arrest
RCC: Renal cell carcinoma
TT: Tumor thrombus
VVB: Veno-venous bypass
OS: Overall survival
DFS: Disease-free survival
DSS: Disease-specific survival
SH: Suprahepatic
RH: Retrohepatic
IH: Infrahepatic
IC: Intracardiac
VVB: Veno-venous bypass
MS: Median sternotomy
MA: Minimal access
SVC: Superior vena cava
TEE: Transesophageal echocardiography
PRISMA: Preferred Reporting Items for Systematic Reviews and Meta-Analyses
ROBINS-I V2: Risk Of Bias In Non-randomized Studies of Interventions Version 2
GRADE: Grading of Recommendations Assessment, Development, and Evaluation
