# Supplementary material for: Cardiopulmonary bypass versus non-bypass surgery for tumor thrombus extending into the inferior vena cava or right atrium in non-cardiac malignancies: a systematic review and meta-analysis
Source: World J Surg Oncol. 2025 Oct 14;23:368. doi: 10.1186/s12957-025-03956-2 (PMC12522322; doi:10.1186/s12957-025-03956-2)
Supplement: Supplementary file 1 — Supplementary Material 1. [file 12957_2025_3956_MOESM1_ESM.docx]

## **Supplement Annex S1**

## **Search Strategy**

## **PubMed**

("tumor thrombus"[Title/Abstract] OR "neoplastic thrombus"[Title/Abstract])

AND

("inferior vena cava"[Title/Abstract] OR "IVC"[Title/Abstract] OR "right atrium"[Title/Abstract] OR "intracardiac"[Title/Abstract])

AND

("surgical resection"[Title/Abstract] OR "thrombectomy"[Title/Abstract])

AND

("cardiopulmonary bypass"[Title/Abstract] OR "CPB"[Title/Abstract] OR "off-pump"[Title/Abstract])

AND

("renal cell carcinoma"[Title/Abstract] OR "hepatocellular carcinoma"[Title/Abstract] OR "adrenal carcinoma"[Title/Abstract])

**Scopus**
TITLE-ABS-KEY("tumor thrombus" OR "neoplastic thrombus")

AND TITLE-ABS-KEY("inferior vena cava" OR "IVC" OR "right atrium" OR "intracardiac")

AND TITLE-ABS-KEY("surgical resection" OR "thrombectomy")

AND TITLE-ABS-KEY("cardiopulmonary bypass" OR "CPB" OR "off-pump")

AND TITLE-ABS-KEY("renal cell carcinoma" OR "hepatocellular carcinoma" OR "adrenal carcinoma" OR "non-cardiac malignancy")

**EMBASE**

('tumor thrombus':ti,ab OR 'neoplastic thrombus':ti,ab)

AND ('inferior vena cava':ti,ab OR ivc:ti,ab OR 'right atrium':ti,ab OR intracardiac:ti,ab)

AND ('surgical resection':ti,ab OR thrombectomy:ti,ab)

AND ('cardiopulmonary bypass':ti,ab OR cpb:ti,ab OR 'off-pump':ti,ab)

AND ('renal cell carcinoma':ti,ab OR 'hepatocellular carcinoma':ti,ab OR 'adrenal carcinoma':ti,ab OR 'non-cardiac malignancy':ti,ab)

**Cochrane Library**

("tumor thrombus" OR "neoplastic thrombus")

AND ("inferior vena cava" OR "IVC" OR "right atrium")

AND ("surgical resection" OR "thrombectomy")

AND ("cardiopulmonary bypass" OR "CPB" OR "off-pump")

AND ("renal cell carcinoma" OR "hepatocellular carcinoma")

**Web of Science**

TS=("tumor thrombus" OR "neoplastic thrombus")

AND TS=("inferior vena cava" OR "IVC" OR "right atrium" OR "intracardiac")

AND TS=("surgical resection" OR "thrombectomy")

AND TS=("cardiopulmonary bypass" OR "CPB" OR "off-pump")

AND TS=("renal cell carcinoma" OR "hepatocellular carcinoma" OR "adrenal carcinoma" OR "non-cardiac malignancy")

**Google Scholar**

"tumor thrombus"

AND "IVC"

AND "cardiopulmonary bypass"

AND "thrombectomy"

AND "renal cell carcinoma"
